# Cinnamtannin B-1 Prevents Ovariectomy-Induced Osteoporosis *via* Attenuating Osteoclastogenesis and ROS Generation

**DOI:** 10.3389/fphar.2020.01023

**Published:** 2020-07-10

**Authors:** Meng Li, Li Hao, Lei Li, Lei Liu, Guang Chen, Wei Jiang, Wei Xu, Chen Zhu, Gang Yao, Shiyuan Fang

**Affiliations:** ^1^Department of Orthopaedics, The First Affiliated Hospital of USTC, Division of Life Sciences and Medicine, University of Science and Technology of China, Hefei, China; ^2^Department of Oncology, The First Affiliated Hospital of USTC, Division of Life Science and Medicine, University of Science and Technology of China, Hefei, China; ^3^Department of Orthopaedics, The Fourth Affiliated Hospital of Anhui Medical University, Hefei, China

**Keywords:** steoporosis, osteoclast, reactive oxygen species, Cinnamtannin B-1, NF-κB pathway

## Abstract

Osteoporosis (OP) is one of the common bone metabolic diseases that endangers postmenopausal women and the elders. Both excessive bone resorption caused by osteoclast over-activation and increased oxidative stress are associated with osteoporosis. Cinnamtannin B-1 (CB-1) is considered as a high-valued plant extract monomer due to its antioxidant properties. However, the mechanism of CB-1 impacts on reducing oxidative stress, inhibiting the production of reactive oxygen species (ROS) and osteoclast differentiation and preventing ovariectomy-induced osteoporosis are still unclear. In this study, the effects of CB-1 on nuclear factor κB (RANKL)-induced osteoclasts formation and differentiation *in vitro* and the potential therapeutic effect on ovariectomy (OVX)-induced osteoporosis *in vivo* are investigated. CB-1 was found to inhibit osteoclast formation and bone resorption function in a dose-dependent manner, and it inhibited specific genes related to osteoclast as well. Micro-CT and histopathological staining showed that CB-1 can effectively prevent OVX-induced osteoporosis. In addition, CB-1 treatment can effectively inhibit the production of reactive oxygen species (ROS) *in vivo* and *in vitro*. Mechanistically, CB-1 inhibits the activation of osteoclasts by inhibiting the activation of the NF-κB signaling pathway. In conclusion, CB-1 would be able to be used as a promising new drug strategy to inhibit RANKL-induced osteoclastogenesis and prevent ovariectomy-induced osteoporosis.

## Introduction

Bone metabolism is a basic process to maintain the continuous renewal of bone tissue, and this process is completed by bone reconstruction (Debnath et al., 2018). Normal bone reconstruction is a dynamic balance between osteoblast (OB) -mediated bone formation and osteoclast (OC) -mediated bone resorption (Mediero and Cronstein, 2013). Prolonged and excessive bone resorption can break this balance leading to diseases such as osteoporosis (Vives et al., 2011; Wang and Grainger, 2012). There are more than 200 million osteoporosis patients around the world, and more than 8.9 million fractures are caused by osteoporosis each year (Vellucci et al., 2014). Currently, strategies for the treatment of osteoporosis mainly involve anti-bone resorption drugs and bone formation promoting drugs, such as bisphosphonates and parathyroid hormones, respectively (Abrahamsen et al., 2020; Altieri et al., 2020; Dole et al., 2020; Ma et al., 2020). Although these drugs are widely used in the treatment of osteoporosis, many of their adverse reactions limit their use (Wolf et al., 2020). For example, long-term use of bisphosphonates cause atrial fibrillation, jaw necrosis, and severe inhibition of bone turnover (Rollason et al., 2016). Therefore, it is urgent to find a new drug that can be used for a long time without serious side effects to prevent osteoporosis.

Osteoclasts are derived from hematopoietic stem cells and differentiate into mature osteoclasts through monocyte/macrophage precursors (Yamazaki et al., 2001). Osteoclasts are characterized by clear areas and wrinkled boundaries, and bone resorption caused by their excessive activation plays a key role in osteoporosis (Park-Min, 2019). The receptor activator of NF-κB ligand (RANKL) is a type II transmembrane protein from the TNF superfamily, which is essential for the formation and activation of osteoclasts (Wang et al., 2018). The combination of RANKL and RANK results in the activation of a series of key transcription factors, such as NF-κB and activated T cell cytoplasm 1 (NFATc1), regulating the expression of osteoclast-related genes, such as tartrate-resistant acid phosphatase (TRAP), ca-thepsin K (CTSK), and matrix metalloproteinase 9 (Mmp9), subsequently leading to the formation and activation of mature multinucleated osteoclasts (Shi et al., 2020). Activation of mature multinucleated osteoclasts eventually resulted in excessive bone resorption leading to bone loss diseases such as osteoporosis. Therefore, the inhibition of RANKL-induced osteoclast activation is considered an important strategy for the treatment of osteoporosis.

Estrogen deficiency has been considered as the main cause of osteoporosis, however, single estrogen supplementation treatment did not directly improve the bone loss due to estrogen deficiency induced osteoporosis (Xu et al., 2020). Therefore, it is necessary to find a new therapeutic target for osteoporosis. More and more studies reported that reactive oxygen species (ROS) may be an important cause of osteoporosis, and its induced oxidative stress plays an important role in osteoporosis (Thummuri et al., 2017). In addition, it was found that ROS stimulated by RANKL play a crucial role in the differentiation of bone marrow-derived macrophages (BMMs) into osteoclasts (Goettsch et al., 2013). Under the influence of factors such as estrogen deficiency or aging, the continuous increase of ROS in the body promotes the activation of osteoclasts and cause a decrease in bone mass and changes in bone structure (Goettsch et al., 2013; Hyeon et al., 2013). Therefore, the inhibition of the intracellular ROS generation may be another valuable strategy for the treatment of osteoporosis.

As nature trimeric proanthocyanidin, Cinnamtannin B-1 (CB-1) is originally isolated from the bark of *Cinnamomum zeylanicum* and only found in a limited number of plants, including *Linderae umbellatae* and *Laurus nobilis* (Wen et al., 2015; Gonzalez de Llano et al., 2019). CB-1 has been mostly studied for its ability to inhibit platelet aggregation and potentiate the action of insulin, likely due to its antioxidant properties and inhibition of Ca^2+^ mobilization (Wang et al., 2014; Panickar et al., 2015). However, the effect of CB-1 on osteoclast activation and post-menopausal osteoporosis is unclear and needs further investigation.

In the current study, we demonstrated that CB-1 can inhibit RANKL-induced osteoclast activation and bone resorption by inhibiting ROS and NFATc1 expression. In addition, CB-1 is able to prevent ovariectomy (OVX) -induced osteoporosis mouse model *in vivo*.

## Materials and Methods

### Drugs and Reagents

Cinnamtannin B-1 [C_45_H_36_O_18_; MW: 864.7; purity >95% (HPLC)] was obtained from Enzo Life Sciences, Inc. (New York, NY, USA), and was dissolved in DMSO (Sigma-Aldrich, USA). Recombinant RANKL and M-CSF were purchased from R&D Systems (Minneapolis, USA). Primary antibodies to phospho-NF-κB p65 (#3033), phospho-IκBα (#2859), NF-κB p65 (#8242), IκBα (#4814), NFATc1 (#8032S), and c-fos (#2250S) were purchased from Cell Signaling Technology (Boston, USA). Anti-rabbit and anti-mouse HRP-conjugated secondary antibodies were obtained from Multi Sciences (Shanghai, China). A counting kit-8 (CCK-8) was purchased from ApexBio (Boston, USA).

### Cell Extraction and Osteoclast Differentiation

The bone marrow macrophages (BMMs) were isolated from 6 to 8 weeks C57BL/6 mice using the methods approved by the Animal Ethics Committee of Anhui Provincial Hospital Affiliated of Anhui Medical University. Briefly, the bone marrow suspensions were flushed out from the dissected femurs and tibias of mice with a cold medium using a 23-G needle syringe. The suspended cells were seeded T75 flasks (10 × 10^6^ cells per flask) and maintained in α-MEM containing 100 U/ml penicillin/Streptomycin, 10% FBS, and 30 ng/ml M-CSF. After the cells were confluent, they were removed from the flask after trypsinization (NCM Biotech, Suzhou, China), and then seeded into a 24-well plate (NEST Biotechnology, China) at a density of 8 × 10^4^ cells per well with culture medium overnight. The next day, BMMs were stimulated with RANKL at the concentration of 50 ng/ml and the presence different concentrations of CB-1, and then medium replaced every 2 days until osteoclasts formed. After 5 days the cells were then fixed with 2.5% glutaraldehyde in phosphate-buffered saline (PBS) for 10 min and stained for tartrate-resistant acidic phosphatase (TRAP) activity. The TRAP staining was performed using a commercial TRAP kit (#387A, Sigma-Aldrich). TRAP positive multinucleated cells (MNCs) were scored as osteoclast-like (OCL) cells if they had three or more nuclei.

Meanwhile, to examine osteoclast function, BMMs were seeded into a 24-well bone resorption plate (OAP, Corning, New York, USA) at a density of 8 × 10^4^/well in triplicate. The BMMs were induced into osteoclasts in induction medium and different concentrations of CB-1 for 5 days. The plates were subsequently washed by sonication until cells were completely disrupted and removed from the bottle of plates. The visual images of resorption pits were snapped at the magnification of 5× using an inverted microscope (Zeiss) and Image J software was used to quantify the area of bone resorption.

### Cell Proliferation and Cytotoxicity

BMMs cells (4 × 10^3^ cells) were seeded on three 96-well plates to detect the cytotoxicity of CB-1. After incubation at 37°C for 24 h, cells were treated with CB-1 (0, 0.01, 0.1, 1, 10, 20, 50, 100 μM) at different mass concentrations for 24 h. After the cell has been cultured for a specified time, change the cell culture medium, add fresh medium containing 1/10 cell CCK-8 reagent to each well and incubate at 37°C for 2 h, and then detect each well at a wavelength of 490 nm on a full-wavelength plate reader (SuPerMax 3000 AL, China). The entire experimental process was performed according to the instructions of the CCK-8 kit.

### Immunofluorescence Staining of F-Actin Belts and p65

BMMs were seeded in 24-well plates at a concentration of 8 × 10^4^ cells per well and stimulated with stimulating medium (50 ng/ml RANKL, 30 ng/ml M-CSF) for 5 days. After osteoclasts were formed, the cells were fixed with 4% paraformaldehyde (PFA) for 15 min, then washed three times with PBS, and permeated with 0.1% Triton X-100 for 10 min. Cells were then blocked with 2% BSA for 1 h and stained with Rhodamine-Phalloidin (Invitrogen, USA) for 1 h. Nuclei were then stained with 4’,6-diamidino-2-phenylindole (DAPI) for 10 min. The cells were washed three times with PBS for fluorescence microscopy imaging. In addition, p65 antibody (abcam, USA) was used to detect the expression and localization of p65 protein. Unlike f-actin staining, cells were incubated with primary antibodies 4°C overnight and then incubated with secondary antibodies conjugated with Alexa Fluor-488 (abcam, USA) at room temperature for 2 h. Finally, the cells were washed three times with PBS for fluorescence microscopy imaging. ImageJ software (NIH, Bethesda, Maryland) was used to measure F-actin size and nuclei.

### RNA Isolation and Real-Time PCR Analysis of Gene Expression

BMMs were seeded in a six-well plate (4 × 10^5^ cells per well) and cultured with stimulating medium in the presence or absence of CB-1 for 5 days to form osteoclasts. The total RNA was extracted from the cells after adding TRIzol reagent (Beyotime, China). cDNA was synthesized from 2 μg of total RNA using a Prime Scriptreverse transcriptase Master Mix kit (TaKaRa, Japan). Quantitative gene analysis was performed using a Universal SYBR Green Supermix (BIO-RAD, USA). The primers used are presented in **Table S1**. GAPDH was used as an internal control gene. The differences in the amount of total cDNA were normalized to the glyceraldehyde 3-phosphate dehydrogenase gene, which served as an endogenous control.

### Luciferase Reporter Assays of NF-κB, NFATc1, and Nrf2-ARE

The RAW264.7 cell line (ATCC, Manassas, Virginia, USA) was stably transfected with NF-κB, NFATc1 and and Nrf2-ARE luciferase reporter gene constructs [36], and then seeded in 48-well plates at the concentration of 5 × 10^4^ cells per well. Cells were cultured overnight and then pre-treated with CB-1 (10 μM) for 4 h and stimulated with RANKL for 24 h. After stimulation, cells were lysed using luciferase lysis buffer and luciferase activities were measured using a dual-luciferase reporter assay system.

### Western Blot Analysis

RAW264.7 cells were reseeded in six-well plates (5 × 10^5^ cells per well) and stimulated with 50 ng/ml RANKL and different concentrations of CB-1. Then, radioimmunoprecipitation (RIPA) lysis buffer was used to lyse cells to harvest protein. Equal amounts of proteins were separated using SDS-polyacrylamide gel electrophoresis and then transferred to nitrocellulose membranes. The membranes were blocked with QuickBlock™ blocking buffer (Beyotime) before incubation with primary antibodies at 4°C. After overnight shaking, the membranes were washed with TBS-Tween and then incubated with horseradish peroxidase (HRP)-conjugated secondary antibodies. Enhanced chemiluminescence (ECL; Sigma-Aldrich) was used to detect the antibodies, and the relative gray level was analyzed using Image Lab software version 3.0 (Bio-Rad).

### Measurement of Intracellular ROS Activities

According to the instructions (#S0033S, Beyotime Biotechnology, China), 6-carboxy-2’, 7’-dichlorodihydrofluorescein diacetate (carboxy-H2DCFDA) dye was used to study intracellular ROS activity. BMMs was seeded in 24-well plates at a concentration of 8 × 10^4^ cells per well. RANKL and different concentrations of CB-1 were added to the culture medium for 48 h. Then, the culture medium and add 500 μl of diluted DCFH-DA was removed. Incubate in a 37°C cell incubator for 20 min. The cells were washed three times with serum-free cell culture medium to fully remove the DCFH-DA that did not enter the cells. The intracellular ROS activity was measured by an inverted fluorescence microscope and the fluorescence intensity was measured by image J.

### Ovariectomy (OVX)-Induced Osteoporosis Mouse Model

All *in vivo* experiments were performed with the approval of the Animal Ethics Committee of Anhui Provincial Hospital Affiliated of Anhui Medical University. Thirty 10-week-old female C57BL/6 mice (18 ± 1.5 g) were separated randomly into three groups (n = 10/group): Sham group (ovaries were only exteriorized but not resected), OVX group (bilateral ovariectomy was carried out to induce osteoporosis), and OVX+CB-1 group (bilateral ovariectomy was carried out to induce osteoporosis + 20 mg/kg CB-1 treatment). All animals were under 2% pentobarbital sodium anesthesia during the operations, and all mice were allowed to recover for 6 days. Then, the mice in the CB-1-treated group were administered an intragastric injection of CB-1 five times per week for 5 weeks. The mice in the sham group and OVX group were intragastric injected with PBS as a control. Before the mice were sacrificed, blood was collected from each mouse for hematology and blood biochemical analysis.

### Micro-CT and Bone Histomorphometry Analysis

After fixation in 10% neutral buffered formalin for 24 h, mouse femurs were analyzed by a Skyscan 1176 micro-CT (Bruker micro-CT, Kontich, Belgium). The left femurs (n = 6 for each group) were scanned using the following settings: 9 μm equidistant resolution, 50 kV, and 200 μA energy X-ray. The images were then reconstructed to perform three-dimensional (3D) histomorphometric analysis with NRecon software (Bruker micro-CT, Kontich, Belgium). SkyScan software was used to analyze bone mineral density (BMD, mg/mm^3^), bone volume (BV, mm^3^), bone volume per tissue volume (BV/TV, %), connectivity density (Conn.Dn, 1/mm^3^), trabecular number (Tb.N, 1/mm), trabecular separation (Tb.Sp, mm), and trabecular thickness (Tb.Th, mm) respectively.

Following micro-CT analysis, all femurs were decalcified in 10% ethylenediaminetetraacetic (EDTA, Sigma-Aldrich) at room temperature for 21 days. Femurs were then processed through ethanol and xylene into wax, embedded into paraffin blocks and sectioned on a microtome at a thickness of 5µm. Hematoxylin and eosin (H&E) and TRAP staining were performed. Bone histomorphometric analyses were performed using BIOQUANT OSTEO software (Bioquant Image Analysis Corporation, Nashville, TN, USA). Section images were acquired using an Axiovert 40C optical microscope (Zeiss, Germany).

### Immunohistochemical Staining *In Vivo*

In brief, antigen retrieval with hyaluronidase for 1 h at 37°C and pepsin for 25 min at room temperature was performed after dewaxing and gradient hydration. Then, the sections were incubated with primary antibodies against CTSK (1:200), NFATc1 (1:200) overnight at 4°C, after which they were blocked with secondary antibody homologous serum for 30 min. Next, the sections were counterstained with hematoxylin after incubation with the appropriate secondary antibodies and a Horseradish Peroxidase Color Development Kit (Beijing Solarbio Science & Technology Co., Ltd.). IHC-positive cells were dark brown and were distributed mainly on the surface of the trabeculae and counted using Image Pro-Plus 6.0 software (Media Cybernetics, Bethesda, USA) and bone histomorphometric analysis was performed using BIOQUANT OSTEO software (Bioquant Image Analysis Corporation, Nashville, TN, USA).

For *in vivo* ROS fluorescence detection, the bone tissue specimens just removed were fixed in 10% formalin solution at 4°C for 4 h. Next, add an appropriate amount of tissue OCT-freeze medium to immerse the tissue, and then make a tissue block by quick freezing of liquid nitrogen. Finally, make a 5 μm slice on a constant temperature cryostat and air dry at room temperature, then 0.3% Triton X-100. After 10 min of permeation, add appropriate amount of dihydroethidium (DHE, #S0063, Beyotime Biotechnology, China) dropwise and incubate at 37°C for 1 h. The nuclei were stained with DAPI for 30 min. Finally, the cells were washed three times with PBS for fluorescence microscopy imaging. The fluorescence intensity was measured by image J.

### Statistical Analysis

Data were presented as mean ± SD. Statistical significance was determined using paired t-tests or by one-way analysis of variance with Tukey’s multiple comparison tests. Probability values were considered statistically significant at *p* < 0.05.

## Results

### CB-1 Inhibited RANKL-Induced Osteoclastogenesis *In Vitro*

**Figure 1:**
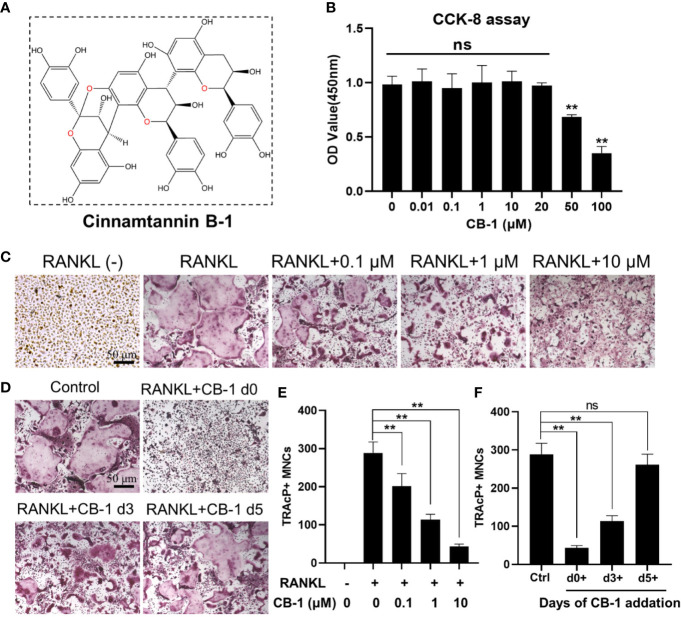
CB-1 suppressed RANKL-induced osteoclastogenesis. **(A)** The chemical structure of CB-1. **(B)** CCK-8 analysis was performed with various concentrations of CB-1 in BMMs for 24 h. **(C)** Representative images of TRAP staining showing that CB-1 inhibited osteoclastogenesis dose-dependently. After pre-treatment with various concentration of CB-1 (0, 0.1, 1, and 10 μM) for 4 h, BMMs were stimulated with 50 ng/ml RANKL for 5 days. **(D)** After pre-treatment with 10 μM CB-1 for 4 h, BMMs were stimulated with 50 ng/ml RANKL and 10 μM CB-1 for the indicated days during osteoclastogenesis. **(E)** Quantification of TRAP-positive multinucleated cells (nuclei ≥3). **(F)** Quantification of TRAP-positive multinucleated cells showing that CB-1 inhibited osteoclastogenesis in different time periods. All bar graphs are presented as mean ± SD. **p < 0.01, ns, no significance, compared with control group, n = 3 per group.

The chemical structure of CB-1 is presented in **Figure 1A**. In order to eliminate the cytotoxic effect of CB-1 on BMMs, we performed CCK-8 cell proliferation and cytotoxicity assay to identify cell viability at first. As shown in **Figure 1B**, CB-1 concentrations ranging from 1 to 20 μM were not cytotoxic to BMMs. Next, BMMs were cultured to investigate the effects of different concentrations of CB-1 on RANKL-induced osteoclast formation. TRAP staining showed that CB-1 inhibited osteoclast formation in a dose-dependent manner (**Figures 1C, E**). In addition, to determine whether the effect of CB-1 on osteoclast differentiation is time-dependent, a time course experiment was performed. Results showed that CB-1 significantly inhibited RANKL-induced osteoclast formation in the early stages of osteoclast differentiation (**Figures 1D, F**). Given the observed data, it is clear that CB-1 is able to inhibit osteoclast formation *in vitro*.

### CB-1 Suppressed Osteoclastic Bone Resorption and Gene Expression

**Figure 2:**
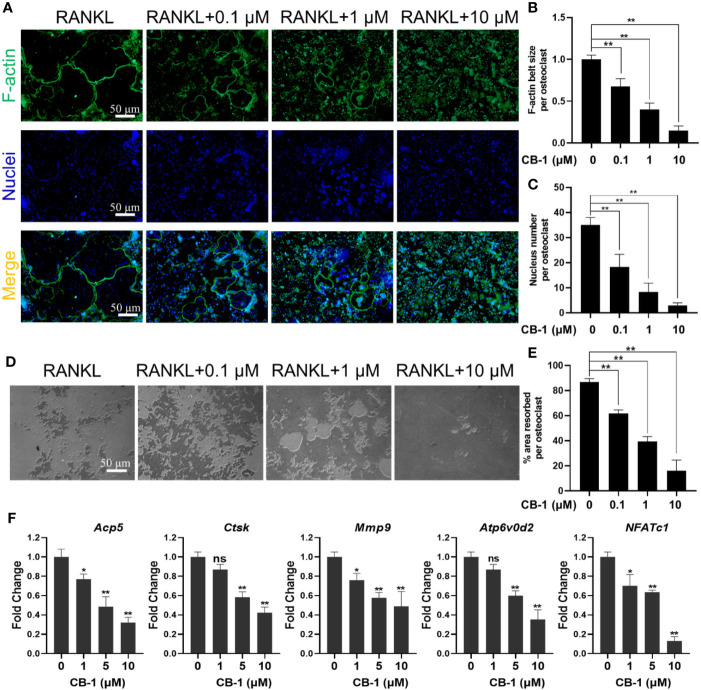
CB-1 inhibited F-actin rings formation, bone-resorbing function, and osteoclast-specific genes expression. **(A)** Representative images showing the impaired podosome belts formation in osteoclasts treated with CB-1. F-actin (green) and nuclei (blue) staining of osteoclasts were observed by fluorescence microscope. **(B)** Quantification of F-actin belt size per osteoclast. **(C)** Quantification of nucleus number per osteoclast. **(D)** Representative images showing the hydroxyapatite resorption in each group. BMMs were plated on bone resorption plates and cultured with induction medium containing 50 ng/ml RANKL together with various concentrations of CB-1 (0, 0.1, 1, and 10 μM) for 5 days. Resorption pits were observed by an inverted microscope. **(E)** Quantification of pits formation area. **(F)** qPCR analysis of osteoclast-specific genes expression of Acp5, Ctsk, Mmp9, Atp6v0d2, and NFATc1 in BMMs stimulated with RANKL for 5 days in the presence of CB-1 (n = 3 per group). All bar graphs are presented as mean ± SD. *p < 0.05, **p < 0.01, ns, not statistically significant, compared with non-treatment group.

Herein, the effort of CB-1 on the formation of osteoclast F-actin belts was investigated to verify its function on bone resorption. The size and nucleus of osteoclasts by staining the cytoskeleton and nucleus was examined. It is found that the F-actin belts of mature osteoclasts treated with CB-1 was dysplastic (**Figures 2A–C**). Along with the inhibitory effect on the F-actin belts, the bone resorption area of Osseo Assay plate after CB-1 treatment decreased as the concentration of CB-1 increased (**Figures 2D, E**). In addition, the effect of CB-1 on the expression of genes was tested as well, which is related to osteoclast differentiation. It is found that Ctsk, Mmp9, Acp5, ATP6V0d2, and NFATc1 genes were significantly down-regulated after CB-1 treatment (**Figure 2F**). These data indicated that CB-1 is capable of suppress the bone resorption function of osteoclasts *in vitro*.

### CB-1 Inhibited Osteoclast Formation *Via* NF-κB Signaling Pathway

**Figure 3:**
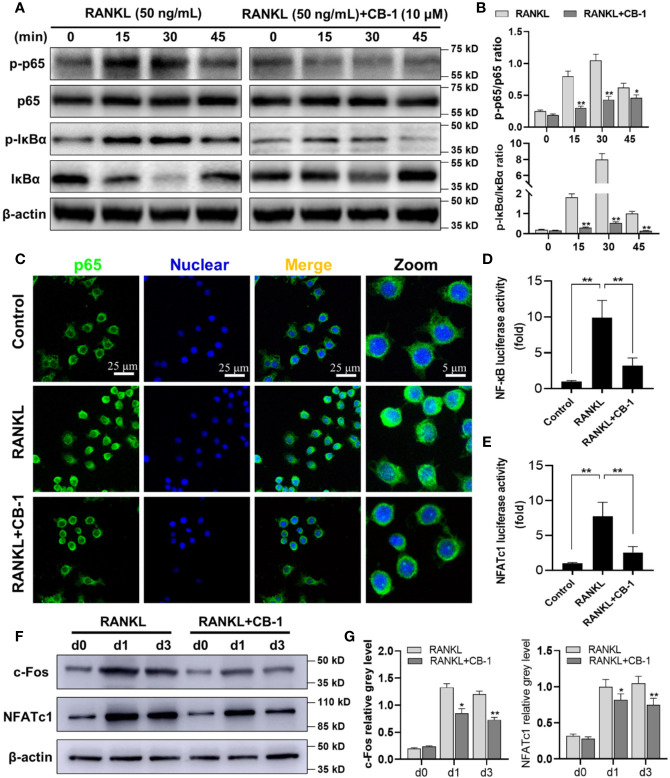
CB-1 interfered with RANKL-induced activation of NF-κB and NFATc1 pathways. **(A, B)** Raw264.7 cells were pretreated with CB-1 (10 μM) for 4 h and then treated with RANKL for 15 to 45 min before lysed in RIPA Buffer. Phosphorylated and total IκBα and NF-κB p65 proteins were detected by specific antibodies. RANKL induced the phosphorylation of IκBα and NF-κB p65, which was significantly inhibited by CB-1. **(C)** After treated with or without CB-1 (10 μM), RAW264.7 cells were stimulated by 50 ng/ml RANKL for 1 h and then stained for NF-κB p65 antibody and secondary antibody with FITC. Immunofluorescence demonstrated that NF-κB p65 protein was translocated into nucleus upon RANKL treatment, and this nuclear translocation was inhibited by CB-1. **(D, E)** NF-κB and NFATc1-binding site-specific luciferase reporter assay showed that RANKL promoted NF-κB and NFATc1 transcription activity, which were suppressed by CB-1. **(F)** After pretreatment with or without CB-1 (10 μM) for 4 h, the cells were cultured in induction medium for 0, 1, or 3 days. Cells were then collected and lysed for western blot analysis. **(G)** The relative gray levels of corresponding to c-Fos and NFATc1 were quantified and normalized to β-actin using ImageJ software. Data are presented as mean ± SD; *P < 0.05 and **P < 0.01 compared with the control group. Data are representative of at least three independent experiments.

To further study the mechanism by which CB-1 inhibits osteoclast differentiation, the effect of CB-1 on the NF-κB pathway was investigated. **Figures 3A, B** showed that the degradation of IκBα was inhibited upon the treatment with CB-1 (10 μM), comparing with RANKL alone. Meanwhile, phosphorylated p65 was also found to be inhibited by BMMs after the treatment with CB-1 (10 μM) (**Figures 3A, B**). Immunofluorescence staining was used to investigate the effect of CB-1 on the nuclear translocation of p65 in BMMs. The results indicated that the majority of p65 was located in the cytoplasm, but after being induced by RANKL and M-CSF, p65 was phosphorylated and translocated to the nucleus. However, the level of the nuclear translocation of p65 was inhibited by CB-1 (**Figure 3C**). Further, CB-1 reduced NF-κB transcriptional activity induced by RANKL as measured by luciferase reporter gene assay (**Figure 3D**). In addition, we find CB-1 had little inhibitory effect on activation of the ERK, p38 and JNK signaling pathways (**Figure S1**). In conclusion, CB-1 inhibit RANKL-induced NF-κB signaling pathway in differentiation of mature multinucleated osteoclasts.

### CB-1 Suppressed RANKL-Induced NFATc1 Expression

Next, to explore whether CB-1 has an inhibitory effect on NFATc1 and c-Fos expression, we treated BMMs with RANKL with and without CB-1 for 0, 1, and 3 days. It was observed that the NFATc1 and c-Fos protein expression in the CB-1 treatment group were obviously lower than that in the untreated group in 1- and 3-days treatment (**Figures 3E–G**). Moreover, CB-1 reduced NFATc1 transcriptional activity which was induced by RANKL as measured by luciferase reporter gene assay (**Figure 3E**). These findings indicated that CB-1 has the capability to suppress NFATc1 expression mediated by RANKL, which are the key molecules for osteoclastic formation.

### CB-1 Attenuated RANKL-Induced ROS Generation in BMMs

**Figure 4:**
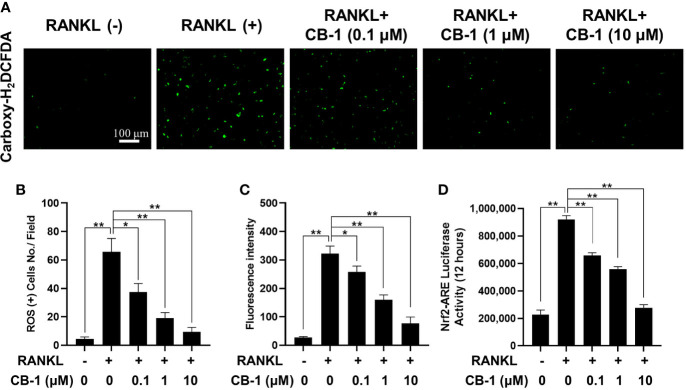
CB-1 suppresses intracellular ROS activity. **(A)** Representative immunofluorescence image of RANKL-induced ROS in the presence or absence of pre-treated CB-1 (10 μM). Intracellular ROS was detected by a carboxy-H2DCFDA dye in the form of highly fluorescent DCF. **(B)** Quantification of DCF fluorescence intensity in an average per cell. **(C)** Quantification of ROS positive cells number per field. **(D)** Oxidative stress was indicated by Nrf2-ARE transcriptional activity and measured by luciferase reporter gene. All bar charts are presented as mean ± SD; n = 3. *p < 0.05 and **P < 0.01 relative to non-treatment group.

In order to study the effect of CB-1 on the intracellular ROS level during RANKL-induced osteoclast differentiation, the CB-1 treatment group was found to prohibit the intracellular ROS responding to RANKL stimulation in a dose-dependent manner without affecting the number of BMMs (**Figures 4A–C**). In another way, after CB-1 treatment, the function of ROS converting DCFH-DA to highly fluorescent DCF was significantly inhibited. Additionally, the effect of CB-1 on ROS-mediated Nrf2-ARE transcriptional activity was investigated by a luciferase reporter assay. With RANKL stimulation, ROS-mediated Nrf2-ARE activity increased over 24 h. However, ARE activity was significantly down-regulated in the presence of CB-1, which was consistent with the observed reduction in ROS levels (**Figure 4D**), suggesting that CB-1 effectively eliminated ROS generation. Combined with the previous results, CB-1 can effectively inhibit RANKL-induced osteoclast differentiation by inhibiting intracellular ROS generation.

### CB-1 Prevents Bone Loss by OVX-Induced *In Vivo*

**Figure 5:**
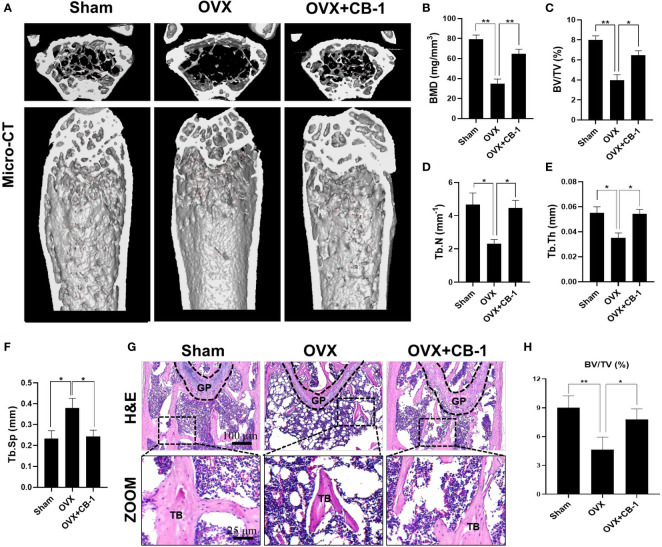
CB-1 inhibited ovariectomized induced bone loss. **(A)** Micro-CT reconstruction images, **(B)** Bone mineral density (BMD, mg/cm^3^), **(C)** Bone volume/tissue volume (BV/TV, %), **(D)** Trabecular number (Tb.N, 1/mm), **(E)** Trabecular thickness (Tb.Th, mm), **(F)** Trabecular separation (Tb.Sp, mm). **(G)** Representative images of H&E staining of decalcified bone sections (n = 5 per group). **(H)** Quantitative analyses of BV/TV in tissue sections (n = 3 per group). *p < 0.05, **p < 0.01 compared with the OVX group. GP, growth plate; TB, trabecular bone.

CB-1 has been confirmed to inhibit osteoclast activation *in vitro*, and then an OVX-model in mice was established to study the potential role of CB-1 in preventing osteoporosis *in vivo*. As well known, estrogen deficiency-mediated bone loss is the basis of human postmenopausal osteoporosis. Based on our research work, no adverse events or deaths were recorded after OVX surgery and CB-1 treatment. The body weight of OVX mice and CB-1 treatment mice were higher than that of the sham controls (**Figure S2**). From the micro-CT three-dimensional (3D) reconstruction (**Figures 5A, B**), comparing with the sham group, the BMD of the OVX group mice was significantly reduced and the bone microstructure changed. Fortunately, CB-1 prevented a large amount of bone loss in the OVX mice. Quantitative analysis showed that bone parameters including BV/TV, Tb.N, and Tb.Th increased, while Tb.Sp and BS/BV decreased in the CB-1 treatment group (**Figures 5C–F**).

**Figure 6:**
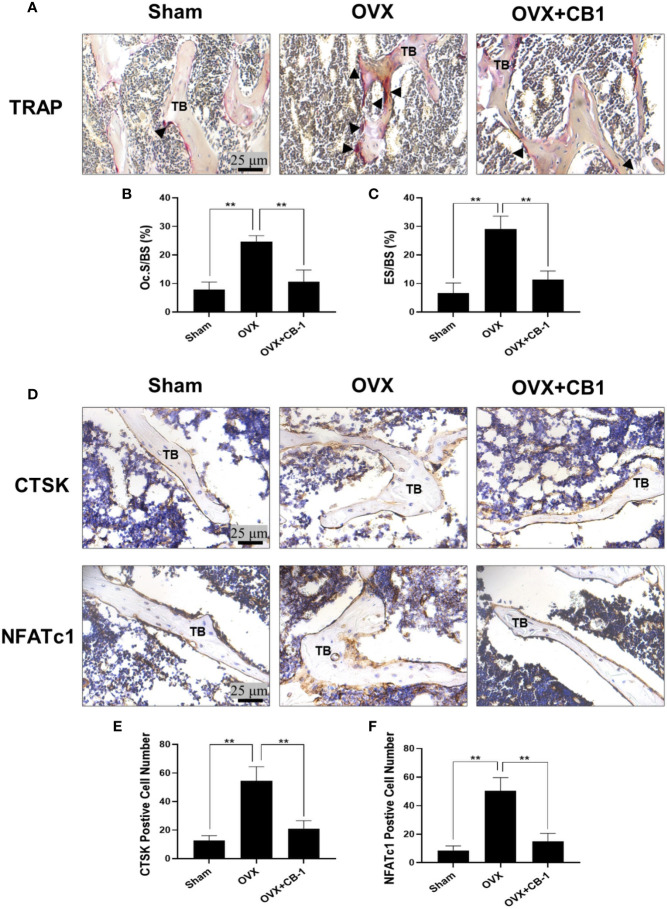
CB-1 reduced osteoclast number and decreased CTSK and NFATc1 levels in OVX mice. **(A)** Representative images of TRAP staining of decalcified bone sections. **(B, C)** Quantitative analyses of Oc.S/B and ES/BS (%) (n = 4 per group). **(D)** Representative immunohistochemical staining for CTSK and NFATc1 of decalcified bone sections. **(E)** CTSK positive cell numbers and **(F)** NFATc1 positive cell numbers were determined (n = 4 per group). All bar graphs are presented as mean ± SD. **P < 0.01 compared with the OVX group. TB, trabecular bone.

Consistently, H&E staining showed that compared with the OVX group, the BS/BV in the CB-1 treatment group maintained well, and the bone loss caused by OVX was significantly reduced (**Figures 5G, H**). TRAP staining represented that, in comparison of the OVX group, the number of osteoclasts and the surface area of osteoclasts on each bone surface were reduced after CB-1 treatment (**Figures 6A–C**). In addition, the expression of osteoclast-related proteins was observed by immunohistochemical staining, and the results demonstrated that the expression of the marker protein CTSK and NFATc1 responsible for osteoclast function was inhibited by CB-1 (**Figures 6D–F**). Again, these results proved that CB-1 can effectively prevent OVX-induced bone loss.

**Figure 7:**
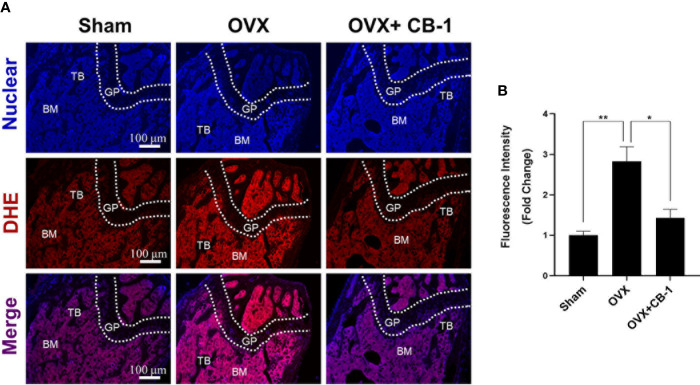
CB-1 treatment reduces ROS production in OVX mice. **(A)** Representative images of bone cryosections showing ROS fluorescence in different groups. **(B)** Quantitative analyses of ROS fluorescence intensity relative to sham group (n = 4 per group). All bar graphs are presented as mean ± SD. *p < 0.05, **P < 0.01 relative to the OVX group. BM, bone marrow; DHE, dihydroethidium; GP, growth plate; TB, trabecular bone.

In addition, since CB-1 exhibits good antioxidant activity *in vitro*, DHE (probe for detecting ROS) was also used to evaluate ROS levels in frozen sections of bone tissue. Due to the apparent increase of ROS fluorescence intensity after OVX, CB-1 significantly reversed ROS production in the bone marrow microenvironment (**Figures 7A, B**). At the same time, frozen sections of liver and kidney tissue staining showed that CB-1 had no hepatorenal toxicity in mice at the given dose (**Figure S3**). Both blood routine and blood biochemistry examinations of CB-1 treated mice showed the same pattern as normal mice, indicating that CB-1 injection did not induce significant hematological toxicity (**Tables S2** and **S3**). In a summary, these data indicated that CB-1 prevents OVX-induced bone loss *in vivo* by eliminating ROS and thereby preventing osteoclast activity.

## Discussion

Bone tissue homeostasis is regulated by a balance between bone formation by osteoblasts and bone resorption by osteoclasts (Karner and Long, 2017). However, under pathological conditions such as estrogen deficiency, tumor necrosis factor (TNF) superfamily ligands and oxidative stress, they all promote the activation of osteoclasts, unbalance bone metabolism and lead to excessive absorption of bone tissue (Tanaka et al., 2005; Kong et al., 2019; Schroder, 2019). Although M-CSF is important factor for osteoclast formation, we found that the CB-1 treatment hardly affects M-CSF/c-Fms signaling pathway (**Figure S4**). During the pathologic process of osteoporosis, reactive oxygen species (ROS) and over activation of osteoclasts may be an important cause of osteoporosis, and its induced oxidative stress plays an important role in osteoporosis (Thummuri et al., 2017). In addition, it was found that ROS stimulated by RANKL play a crucial role in the differentiation of bone marrow-derived macrophages (BMMs) into osteoclasts (Goettsch et al., 2013). Under the influence of factors such as estrogen deficiency or aging, the continuous increase of ROS in the body promotes the activation of osteoclasts and cause a decrease in bone mass and changes in bone structure (Goettsch et al., 2013; Hyeon et al., 2013). Therefore, the inhibition of the intracellular ROS generation may be another valuable strategy for the treatment of osteoporosis. Excessive activation of osteoclasts induces severe osteoporosis (Liu et al., 2019). Osteoclasts are multinucleated giant cells derived from bone marrow mononuclear cells (Takahashi et al., 1995; Iwai et al., 2007). Under the action of chemokines (M-CSF and RANKL), BMMs pass through the blood circulation and reach to bone tissue sites in the state of bone resorption, and they differentiate into osteoclasts (Zhao et al., 2015). In this study, for the first time, we demonstrated that CB-1 inhibits osteoclastogenesis and ROS generation *in vitro* and prevents the development of OVX-induced osteoporotic mice *in vivo*.

NF‐κB signaling pathway plays an instrumental role in transducing and integrating osteoclastogenic signals (Li et al., 2019a; Chang et al., 2020). Evidence indicates that NF‐κB is also an important regulator for bone remodeling, and the blocking of NF‐κB activation inhibits bone loss. Wu et al. observed that hydrogen gas protects against ovariectomy-induced osteoporosis by inhibiting NF-κB activation (Wu et al., 2019). These data indicated that NF‐κB is a promising target for the treatment of osteoporosis. Notably, in this study, the factor that CB-1 suppressed RANKL‐induced IKKα/β phosphorylation was observed, which resulted in the inhibition of NF‐κB signaling activation in osteoclasts. In addition, the nuclear translocation of p65 were also suppressed, further showing that CB-1 suppressed osteoclastogenesis through the inhibition of NF‐κB signaling.

Extensive researches have confirmed that oxidative stress caused by reactive oxygen species (ROS) can lead to imbalance of bone homeostasis and bone metabolism diseases, mainly bone resorption (Agidigbi and Kim, 2019; Almeida and Porter, 2019). In 1990, Garrett added xanthine oxidase that promotes O_2_ production in the experiment and found that it can increase the activity of osteoclasts (Garrett et al., 1990). Notably, this promotion can be weakened by O_2_ scavenging enzyme (SOD), which indicates that ROS involved in osteoclast differentiation. Meanwhile, research has shown ROS may promote the activation of osteoclasts by regulating the activity of osteoclast transcription factors (such as NF-κB, NFATc1) (Jeong et al., 2019; Li et al., 2019b). In our current study, the inhibitory effect of CB-1 on NF-κB and NFATc1 transcriptional activity *in vitro* was demonstrated. ARE is a downstream factor of nuclear factor erythroid-2 related factor 2 (Nrf2), which plays an important role in maintaining intracellular redox homeostasis and resisting cell damage caused by ROS (Guo and Mo, 2020). After RANKL stimulation, an oxidative stress environment is formed (Xu et al., 2010). Nrf2 translocation nucleation combines with Maf protein and ARE to form a heterotrimer, which activates the transcriptional activation of antioxidant enzyme genes (Sanghvi et al., 2019). Interestingly, we not only found that CB-1 inhibited intracellular ROS production during RANKL-induced osteoclastogenesis, but also down-regulated ARE transcriptional activity. This suggests that CB-1 plays a key role in eliminating ROS in osteoclasts.

In this study, the biological function of CB-1 was firstly evaluated by osteoclast differentiation assay, revealing that CB-1 significantly inhibited osteoclast differentiation in a dose-dependent and time-dependent manner. F-actin belts staining and Osseo Assay plate assay further confirmed the inhibitory effect of CB-1 on the osteoclast differentiation and absorption functions. These results suggest that upregulate CB1 could significantly inhibit RANKL-induced osteoclast differentiation *in vitro*. Next, to further explore the basic mechanisms by which CB-1 inhibits osteoclast activation, we observed two classical pathways of osteoclast activation, NF-κB and NFATc1 pathways. Our results indicate that CB-1 disrupts the RANKL-induced activation of NF-κB, leading to the reduction of P65 nuclear translocation and thus reduce the downstream transcription activity of P65. Besides, CB1 can also significantly inhibit the expression of osteoclast differentiation marker protein NFATc1. Considering the crucial role of ROS increasing results in oxidative stress during osteoclast activation in osteoporosis, we further observed the effect of CB1 on ROS regulation in process of osteoclastogenesis. Our findings reveal that CB1 can reduce the production of ROS and thus reduce oxidative stress. Moreover, *in vivo* study, it was observed that CB-1 potently protected against bone loss, according to micro-CT and histopathological analysis. Additionally, TRAP staining and ROS staining further demonstrate that CB-1 treatment decreased the osteoclast number and prevented ROS generation in OVX model mice. Collectively, comprehensive *in vitro* and *in vivo* results, our results demonstrated that CB-1 treatment alleviated OVX-induced bone loss *via* attenuating osteoclastogenesis and ROS generation.

Despite these meaningful findings, the current research has some limitations. First, bone metabolism is a balance between osteoclast absorption and osteoblast formation. Bone formation also plays an important role in the treatment of osteoporosis. So far, whether or not CB-1 can enhance osteoblast formation and thus improve osteoporosis is unknown yet and needs further exploration. This will be investigated in future research. Second, age-related osteoporosis is also common clinically. We chose to use OVX-induced osteoporosis mouse models because it has good characteristics. However, it has been reported that mouse cortical bone does not have the Harvard system, cannot perform Harvard’s reconstruction, and does not cause brittle fracture, so it cannot simulate the effects of cortical bone (Movérare-Skrtic et al., 2014). Therefore, large animal models including sheep or dogs may be more suitable for simulating osteoporosis. The model will be evaluated in future studies. In addition, the effect of CB1 on other signaling pathways such as JNK and Akt-GSK3β pathways during osteoclast activation will also be further studied in following work.

## Conclusions

**Figure 8:**
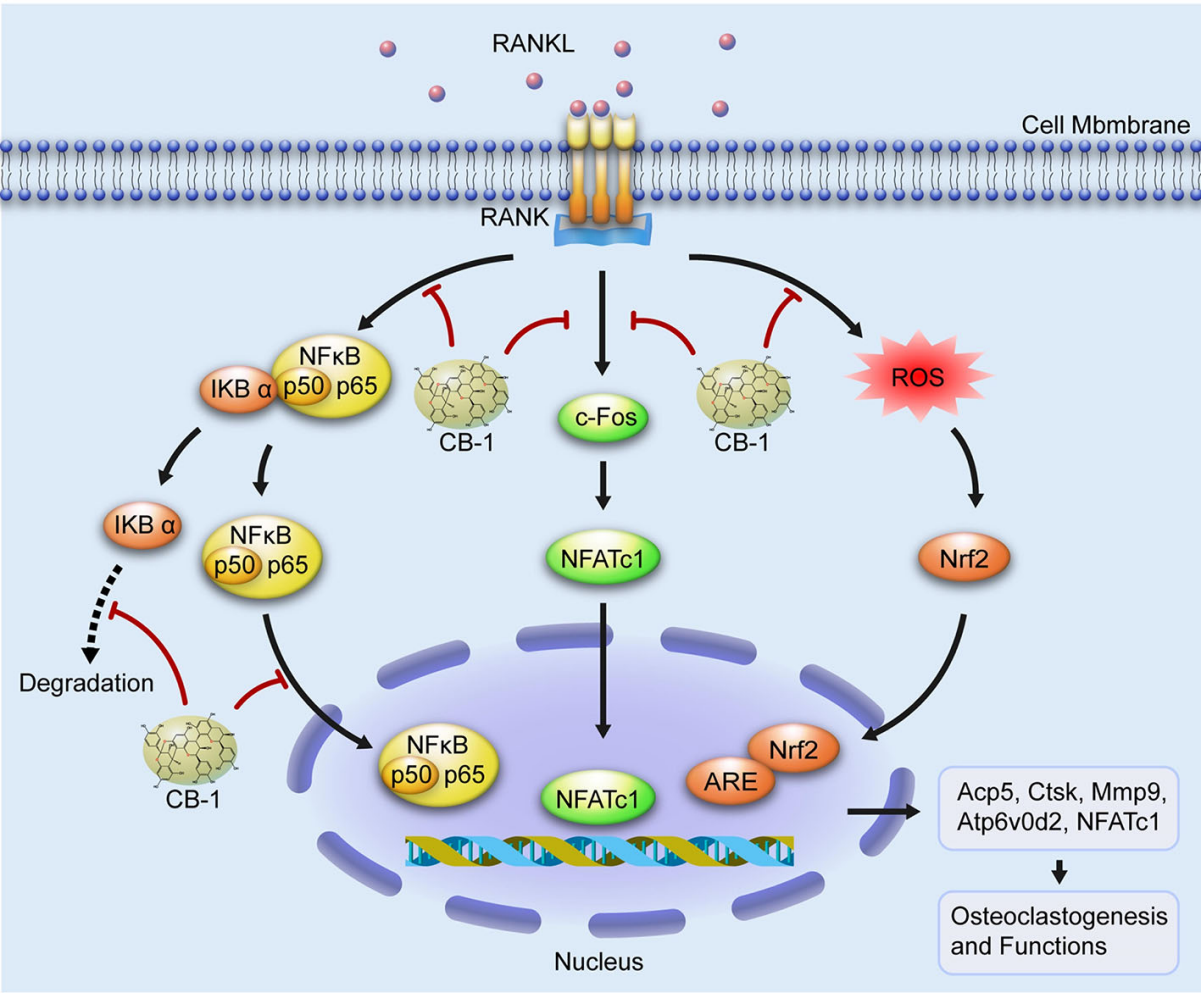
A proposed working scheme for the inhibition of CB-1 on osteoclastogenesis. After RANKL is combined with RANK, NF-κB and NFATc1 are activated and enter the nucleus. Meanwhile, the stimulation of RANKL promotes intracellular ROS generation. Our results indicate that CB-1 can inhibit osteoclast formation and bone resorption by attenuating the activities of NF-κB, NFATc1, and ROS.

In summary, this study shows that CB-1 inhibits osteoclastogenesis and related gene expression and inhibits ROS levels. In addition, this study demonstrates, for the first time, that CB-1 is effective in preventing bone loss caused by OVX mouse models. These results indicate that CB-1 may be a potential therapeutic agent for the treatment of osteoporosis caused by excessive activation of osteoclasts and ROS generation (**Figure 8**).

## Data Availability Statement

The raw data supporting the conclusions of this article will be made available by the authors, without undue reservation, to any qualified researcher.

## Ethics Statement

The animal study was reviewed and approved by the Ethics Committee of Anhui Provincial Hospital Affiliated of University of Science and Technology of China.

## Author Contributions

ML, LH, and LLi contributed to the conception of the work. LLiu, ML, GC, and WJ contributed to the experiments. WX and CZ contributed to the data acquisition. ML and GY wrote the manuscript. LH and SF revised the manuscript. All authors contributed to the article and approved the submitted version.

## Funding

This study was supported by National Natural Science Foundation of China (81373421, 81270650).

## Conflict of Interest

The authors declare that the research was conducted in the absence of any commercial or financial relationships that could be construed as a potential conflict of interest.
