# Supplementary material for: Cinnamtannin B-1 Prevents Ovariectomy-Induced Osteoporosis via Attenuating Osteoclastogenesis and ROS Generation
Source: Front Pharmacol. 2020 Jul 10;11:1023. doi: 10.3389/fphar.2020.01023 (PMC7365944; doi:10.3389/fphar.2020.01023)
Supplement: Supplementary file 1 [file DataSheet_1.docx]

**Supplementary data**


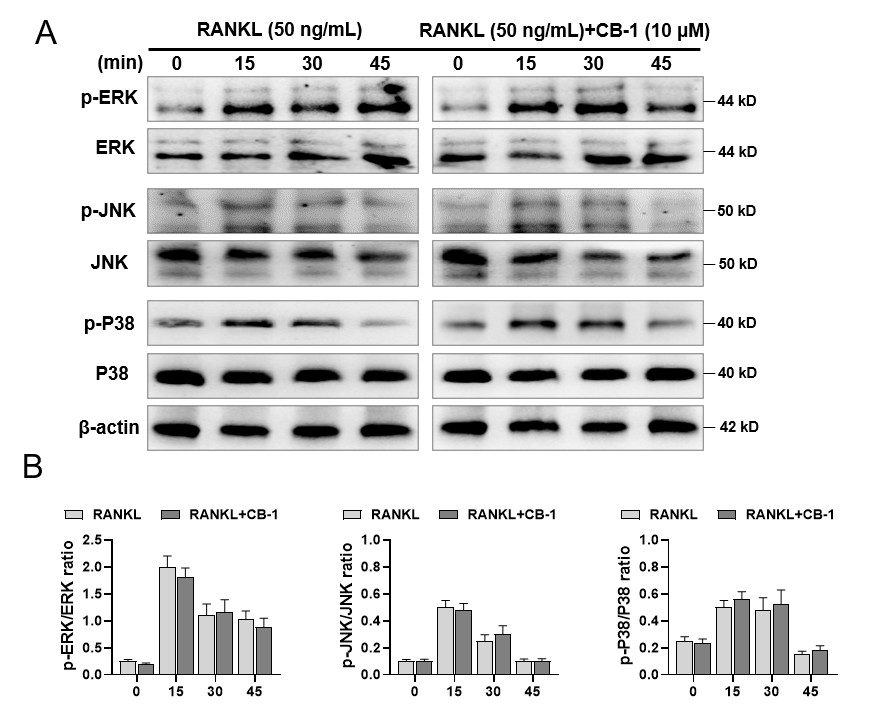


**Fig. S1.** CB-1 had little inhibitory effect of MAPK pathway. RAW264.7 cells were starved for 12 h with or without 10 μM CB-1, and then stimulated with RANKL (50 ng/mL) in the presence or absence of 20 μM CB-1 for 0, 15 and 45 min. Western blotting was used to analyze the expression of total and phosphorylated protein in the above pathways. (A, B) CB-1 had little inhibitory effect on ERK, JNK and P38 pathways. Data are presented as mean ± SD of at least three independent experiments (*p < 0.05, versus the RANKL group).


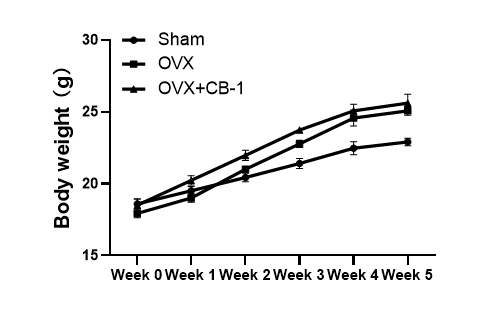


**Fig. S2.** Effects of CB-1 on changes of body weight in OVX mice. Body weight was measured once a week.


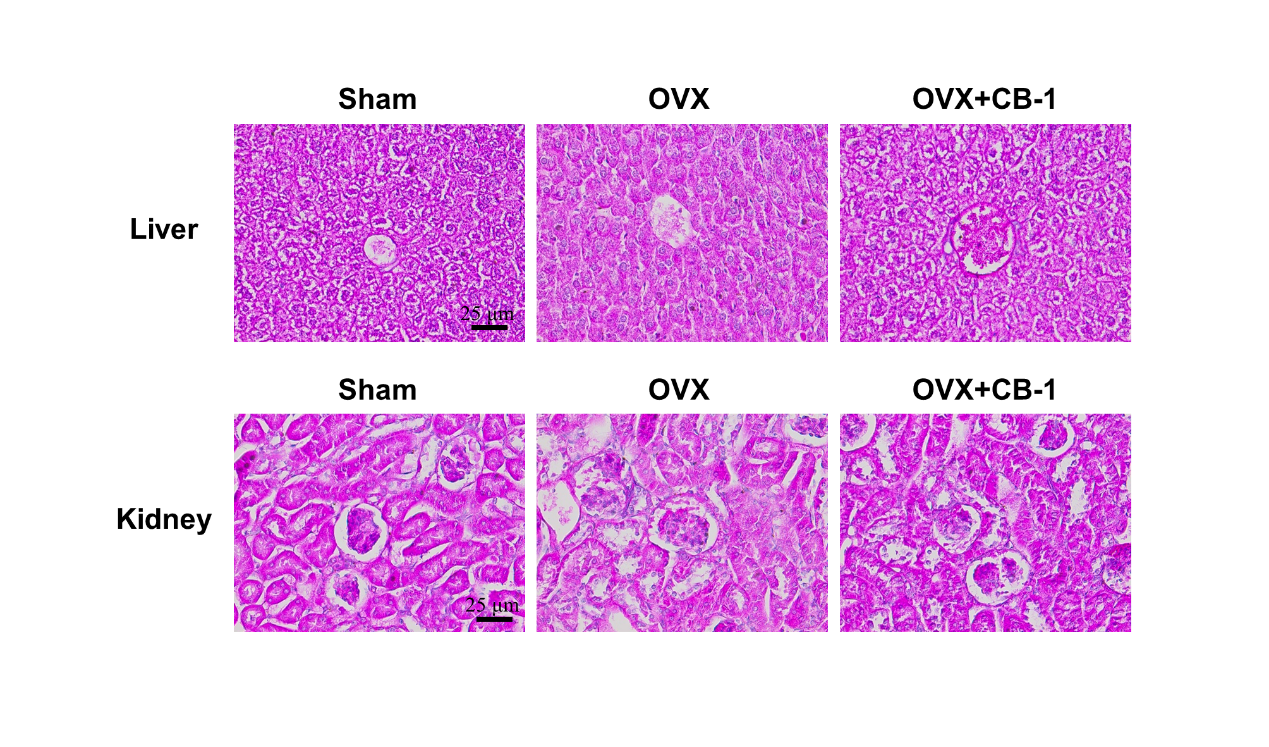


**Fig. S3.** Representative histopathological images of main organs (liver and kidney) from each group of C57BL/6 mice.


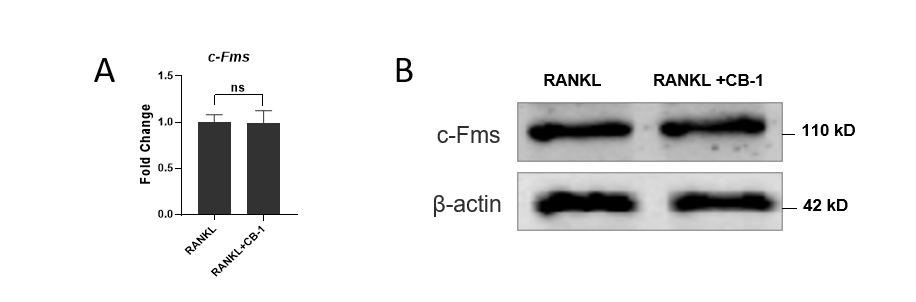


**Fig. S4.** The effect of CB1 for M-CSF signaling. (A) qPCR analysis of osteoclast-specific genes expression of c-Fms in BMMs stimulated with RANKL for 24 h in the presence of CB-1. (B) BMMs were pretreated with CB-1 (10 μM) for 4 h and then treated with RANKL (50 ng/ml) and M-CSF (30 ng/ml) for 24 h before lyzed in RIPA Buffer. Total c-Fms protein were detected by immunoblotting. All bar graphs are presented as mean ± SD. ns = no significance, compared with non-treatment group, n=3 per group.
