# Supplementary material for: Cinnamtannin B-1 Prevents Ovariectomy-Induced Osteoporosis via Attenuating Osteoclastogenesis and ROS Generation
Source: Front Pharmacol. 2020 Jul 10;11:1023. doi: 10.3389/fphar.2020.01023 (PMC7365944; doi:10.3389/fphar.2020.01023)
Supplement: Supplementary file 2 [file Table_1.docx]

**Supplementary data**

**Table S1:**

| **Gene (mouse)** | **Forward primer sequence (5’-3’)** | **Reverse primer sequence(5’-3’)** |
| --- | --- | --- |
| Acp5 | TGTGGCCATCTTTATGCT | GTCATTTCTTTGGGGCTT |
| Ctsk | GGGAGAAAAACCTGAAGC | ATTCTGGGGACTCAGAGC |
| Mmp9 | CGTGTCTGGAGATTCGACTTGA | TTGGAAACTCACACGCCAGA |
| Atp6v0d2 | GTGAGACCTTGGAAGACCTGAA | GAGAAATGTGCTCAGGGGCT |
| NFATc1 | CAACGCCCTGACCACCGATAG | GGCTGCCTTCCGTCTCATAGT |
| GAPDH | CACCATGGAGAAGGCCGGGG | GACGGACACATTGGGGGTAG |

Abbreviations: **Acp5**: acid phosphatase 5, tartrate resistant; **Ctsk**: Cathepsin K; **Mmp9**: matrix metalloprotein 9; **NFATc1**: nuclear factor of activated T cells 1; **GAPDH**: glyceraldehyde 3-phosphate dehydrogenase.
