# Supplementary material for: Cinnamtannin B-1 Prevents Ovariectomy-Induced Osteoporosis via Attenuating Osteoclastogenesis and ROS Generation
Source: Front Pharmacol. 2020 Jul 10;11:1023. doi: 10.3389/fphar.2020.01023 (PMC7365944; doi:10.3389/fphar.2020.01023)
Supplement: Supplementary file 3 [file Table_2.docx]

**Supplementary data**

**Table S2.** Effect of CB-1 on hematology index of mice

| **Index** | **Sham** | **OVX** | **OVX+CB-1** |
| --- | --- | --- | --- |
| WBC（10^9^/L） | 4.14 ± 1.56 | 4.43 ± 1.59 | 4.48 ± 1.39 |
| RBC（10^12^/L） | 12.57 ± 0.46 | 12.38 ± 0.54 | 12.42 ± 0.55 |
| HGB（g/L） | 176.56 ± 5.71 | 174.60 ± 7.21 | 178.80 ± 6.14 |
| HCT（%） | 55.24 ± 2.52 | 55.04 ± 2.12 | 54.20 ± 3.12 |
| MCV（fL） | 45.68 ± 0.45 | 45.46 ± 0.60 | 45.44 ± 0.55 |
| MCH（pg) | 14.41 ± 0.33 | 14.50 ± 0.41 | 14.66 ± 0.26 |
| MCHC（g/L） | 316.80 ± 3.40 | 316.80 ± 3.50 | 316.60 ± 3.63 |
