# Supplementary material for: Cinnamtannin B-1 Prevents Ovariectomy-Induced Osteoporosis via Attenuating Osteoclastogenesis and ROS Generation
Source: Front Pharmacol. 2020 Jul 10;11:1023. doi: 10.3389/fphar.2020.01023 (PMC7365944; doi:10.3389/fphar.2020.01023)
Supplement: Supplementary file 4 [file Table_3.docx]

**Supplementary data**

**Table S3.** Effect of CB-1 on biochemistry index of mice

| **Index** | **Sham** | **OVX** | **OVX+CB-1** |
| --- | --- | --- | --- |
| ALT (U/L) | 29 ± 5 | 31 ± 3 | 30 ± 4 |
| AST (U/L) | 131 ± 47 | 137 ± 39 | 144 ± 43 |
| TP (g/L) | 63 ± 4 | 68 ± 5 | 66 ± 6 |
| ALB (g/L) | 24 ± 1.5 | 23 ± 2.5 | 28 ± 3 |
| ALP (U/L) | 192 ± 21 | 187 ± 27 | 181 ± 33 |
| UREA (mM) | 10 ± 3 | 10 ± 3 | 9 ± 3 |
| CREA (μM) | 10 ± 3 | 10 ± 2 | 9 ± 2 |
